# An open-label feasibility trial of transdermal cannabidiol for hand osteoarthritis

**DOI:** 10.1038/s41598-024-62428-x

**Published:** 2024-05-23

**Authors:** Zeeta Bawa, Daniel Lewis, Paul D. Gavin, Roksan Libinaki, Lida Joubran, Mahmoud El-Tamimy, Greg Taylor, Ryan Meltzer, Miguel Bedoya-Pérez, Richard C. Kevin, Iain S. McGregor

**Affiliations:** 1 The Lambert Initiative for Cannabinoid Therapeutics, The University of Sydney, Sydney, New South Wales Australia; 2 School of Psychology, The University of Sydney, Sydney, New South Wales Australia; 3 Sydney Pharmacy School, The University of Sydney, Sydney, New South Wales Australia; 4 The Daniel Lewis Rheumatology Centre, Melbourne, Victoria Australia; 5 Avecho Biotechnology, Melbourne , Victoria Australia; 6 The NTF Group, Sydney, New South Wales Australia

**Keywords:** Medicinal cannabis, Cannabidiol (CBD), Osteoarthritic pain, Chronic pain, Transdermal, Osteoarthritis, Osteoarthritis

## Abstract

Hand osteoarthritis (OA) is an irreversible degenerative condition causing chronic pain and impaired functionality. Existing treatment options are often inadequate. Cannabidiol (CBD) has demonstrated analgesic and anti-inflammatory effects in preclinical models of arthritis. In this open-label feasibility trial, participants with symptomatically active hand OA applied a novel transdermal CBD gel (4% w/w) three times a day for four weeks to their most painful hand. Changes in daily self-reported pain scores were measured on a 0–10 Numeric Pain Rating Scale (NPRS). Hand functionality was determined via daily grip strength measures using a Bluetooth equipped squeeze ball and self-report questionnaire. Quality of life (QoL) ratings around sleep, anxiety, stiffness and fatigue were also measured. All self-report measures and grip strength data were gathered via smartphone application. Urinalysis was conducted at trial end to determine systemic absorption of CBD. Eighteen participants were consented and 15 completed the trial. Pain ratings were significantly reduced over time from pre-treatment baseline including current pain (− 1.91 ± 0.35, *p* < 0.0001), average pain (− 1.92 ± 0.35, *p* < 0.0001) and maximum pain (− 1.97 ± 0.34, *p* < 0.0001) (data represent mean reduction on a 0–10 NPRS scale ± standard error of the mean (SEM)). A significant increase in grip strength in the treated hand (*p* < 0.0001) was observed although self-reported functionality did not improve. There were significant (*p* < 0.005) improvements in three QoL measures: fatigue, stiffness and anxiety. CBD and its metabolites were detected at low concentrations in all urine samples. Measured reductions in pain and increases in grip strength seen during treatment reverted back towards baseline during the washout phase. In summary, pain, grip strength and QoL measures, using smartphone technology, was shown to improve over time following transdermal CBD application suggesting feasibility of this intervention in relieving osteoarthritic hand pain. Proof of efficacy, however, requires further confirmation in a placebo-controlled randomised trial.

Trial registration: ANZCTR public trials registry (ACTRN12621001512819, 05/11/2021).

## Introduction

Osteoarthritis (OA) is a common, heterogeneous disease of synovial joint cartilage, with inflammatory, biomechanical and genetic factors contributing to its aetiology^1,2^. Hand OA can significantly impact the quality of life (QoL), functionality and economic security of those affected^3^. The Framingham Community cohort study established lifetime prevalence rates of ~ 44% in women and ~ 38% in men^1^. Hand OA can be characterised into erosive hand OA, nodal hand OA and thumb base OA, each with its own phenotypes and risk factors^4,5^. Nonetheless, active hand OA symptoms are similar and include pain, stiffness, a reduction in grip and/or pinch strength, inflammation and bony enlargements known as *nodes*^4,6^.

There are no disease modifying treatments for OA^5^ and therapy focuses on symptom relief and preservation of function^5,7^. As such, the first-line therapy for symptomatically active hand OA involves topical non-steroidal anti-inflammatory drugs (NSAIDs). These are recommended for their superior safety profile compared to oral analgesics^5,8^, but are limited in their ability to provide pain relief^9^. Oral analgesics are prescribed when there is insufficient relief of symptoms with topical medications. However, oral NSAIDs can lead to troublesome gastrointestinal, cardiovascular, renal adverse effects that are amplified in the elderly population^5,8^. Given the narrow range of treatment options for symptomatically active hand OA, there is an urgent need for the development of novel and efficacious treatment options, yet progress in this area has been slow, if not stationary^7^.

Cannabidiol (CBD) is a non-intoxicating constituent of cannabis that has shown efficacy in the treatment of epilepsy, anxiety and psychosis^10–12^. Reviews of the literature suggest only limited available data around the efficacy of CBD in OA, chronic pain^13–15^ and inflammation^16,17^. A 12-week randomised, double-blind, placebo-controlled trial of oral CBD (20–30 mg/day) as an add-on therapy in 136 patients with hand OA or psoriatic arthritis found no significant difference to placebo in the primary outcome measure of pain intensity during the past 24 h^18^. It should be noted, however, that 20–30 mg is a very low oral CBD dose^13^. In a trial of transdermal CBD for OA, adults with knee pain due to OA were treated with Zygel™, a transdermal synthetic CBD gel that was massaged onto the upper arm twice daily for 12 weeks^19^. While the primary endpoint was not reached in this trial (change in the 24-h average worst pain score at Week 12), the secondary endpoints (≥ 30 percent reduction in worst average daily pain scores and a ≥ 20 percent improvement in physical function scores) were met^19^. In a recent trial, the effects of six weeks of topical CBD (10 mg) applied twice a day to the legs were explored in 20 retired elite athletes who experienced chronic pain from acute lower extremity injuries. Self-reported pain was significantly improved including aspects of pain-related disability^20^. Finally, in two recent clinical studies involving COVID-19 and cancer patients respectively, CBD failed to demonstrate anti-inflammatory effects^16,17^. Preclinical studies, however, have demonstrated analgesic and anti-inflammatory effects of CBD in OA and other conditions^21–30^. For example, transdermal CBD gel applied four times daily significantly reduced pain-related behaviours and inflammation in rats with induced arthritic joints without any notable adverse effects^31^. Given this preliminary evidence, further exploration of the feasibility of using transdermal CBD as an intervention in OA is warranted.

Transdermal application has advantages for conditions that only require a localised action of CBD^32^. This route bypasses first-pass metabolism in the liver and gastrointestinal tract^32–34^ and minimises adverse effects and drug-drug interactions associated with oral CBD doses^32^, possibly promoting greater treatment adherence^32,35^. Transdermal treatments generally provide a superior safety profile compared to the oral analgesics used in the management of hand OA^5,36^. Indeed, the first-line therapy for symptomatic hand OA is topical non-steroidal anti-inflammatory drugs (NSAIDs)^5,8,36^, although the reported effectiveness of these products has been less than ideal^9^.

It is important to note that CBD is a highly lipophilic molecule^37^. While it penetrates the upper layers of the stratum corneum with ease, permeation into the deeper, relatively aqueous layers of the dermis is relatively low^34,37^. Consequently, various transdermal permeation enhancers have been trialled to increase CBD bioavailability^34,37–41^. The current study utilised a novel transdermal CBD gel preparation containing a mixture of two forms of phosphorylated vitamin E (i.e., a tocopheryl phosphate mixture (TPM)) as a permeation enhancer. Effects of this CBD formulation on hand pain and functionality in patients with symptomatically active hand OA were examined over a four-week intervention phase. It was hypothesised that treatment with transdermal CBD gel would decrease measures of pain and improve hand functionality.

### Ethics approval

This study was approved by the Bellberry Ethics Committee on 1 February 2022 (Ref: 2021-07-787-A-1) and conducted in accordance with Good Clinical Practice guidelines and the Declaration of Helsinki (1983). All participants provided written informed consent prior to enrolment into the study. This study was registered on the ANZCTR public trials registry (ACTRN12621001512819, 05/11/2021). 

## Method

### Study design

**Figure 1:**
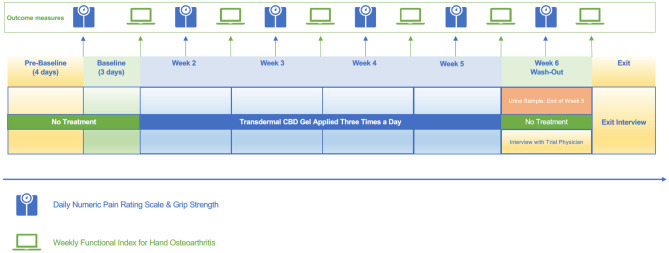
Study flowchart illustrating pre-baseline practise, baseline, treatment and washout phases of the trial.

This was a single-centre, interventional, feasibility trial. An overview of the study design is summarised in the Study Flowchart (Fig. 1). All participants completed the study at the same time. The study involved a one-week run-in phase which included a 4-day “Pre-baseline practise” phase where participants practised data acquisition procedures including use of the device for measuring grip strength. This was followed by a 3-day “Baseline” phase which provided the baseline comparator data for the intervention. During a four-week “Treatment” phase, participants were instructed to apply 0.25 mL of a transdermal 4% w/w CBD gel three times per day (~ 30 mg CBD per day) to the same hand (their most painful hand, that is, the “treated hand”) while leaving the other hand untreated (the “untreated hand”). This is because hand OA does not always present bilaterally^42^, precluding the uniform use of the untreated hand as a control. Participants were advised to base their self-reported outcome measures on the treated hand. At the end of the 4 week Treatment phase, participants underwent a one-week “Washout” phase during which the CBD transdermal gel was ceased while measurement of outcomes continued.

### Participant population

The COVID-19 pandemic and the exploratory nature of the study limited recruitment to up to 20 participants. This sample size was chosen based on the number of long-term patients under the care of the trial physician who would likely meet the eligibility criteria for the trial. Participants met the inclusion criteria if they had painful symptomatic distal interphalangeal (DIP) nodal hand OA affecting at least three joints of the fingers and thumb, where at least one DIP node had 1) a pain severity of at least three on a 0–10 Numeric Pain Rating Scale (NPRS), and 2) pain on most days of the month for at least one month in the last year.

Exclusion criteria were as follows: (1) a history of or current inflammatory arthritis (examples: gout, psoriatic arthritis, and rheumatoid arthritis); (2) medications and/or medical conditions likely to change over the six-week trial phase; (3) prior surgery on the DIP joint(s); (4) pregnancy or lactation; (5) dermatological conditions of the hand; (6) use of cannabis, or cannabis-based products during the last three months; and (7) known allergies or hypersensitivity to cannabis-based products.

### Recruitment, screening and enrolment

Participants with symptomatic hand OA were recruited from an outpatient rheumatology clinic in Victoria, Australia between February to March 2022. The trial physician conducted a thorough medical history of all volunteers and excluded those who noted recent use of cannabis or cannabis-derived products, including CBD. Volunteers were screened on-site and informed of the study risks. Volunteers reviewed the participant information sheet prior to providing written informed consent. Subsequently, the trial physician conducted a mandatory physical examination and medical history check prior to enrolling participants to the study. The trial physician also assisted participants to determine which of their hands would be treated and which would remain untreated. Participants attended a single group training session detailing the study procedures prior to commencing the trial.

### Study treatment

The investigational product was a novel transdermal 4% w/w CBD gel containing tocopheryl phosphate mixture (TPM) that is used as a transdermal permeation enhancer^43^. TPM self-assembles into nanostructures in the presence of water, forming elastic vesicles able to encapsulate lipophilic molecules at high efficiency to increase their solubility in aqueous environments^43–45^. As reported for other highly elastic vesicle systems such as ethosomes^46^ and transfersomes^47–49^, TPM increases the dermal absorption of a variety of drug molecules^43^. Currently, two topical anti-inflammatory gels formulated with TPM (Voveran TPM® and Aquadol TPM Gel®) are marketed in India for the treatment of pain associated with OA^50,51^.

As this was an early phase clinical trial, a compounding pharmacy in Victoria, Australia was used to prepare, package and label the investigational product. As permitted by Australian standards, the extemporaneous preparation of medicines by pharmacists is considered exempt from good manufacturing practice (GMP) licensing^52^. Furthermore, the compounding pharmacy had no role in the design or analysis of the study. Participants were provided with a single 30 mL pump pack and instructed to apply 0.25 mL three times per day (~ 30 mg CBD per day) to the treatment hand during the four-week Treatment phase.

### Data collection

**Figure 2:**
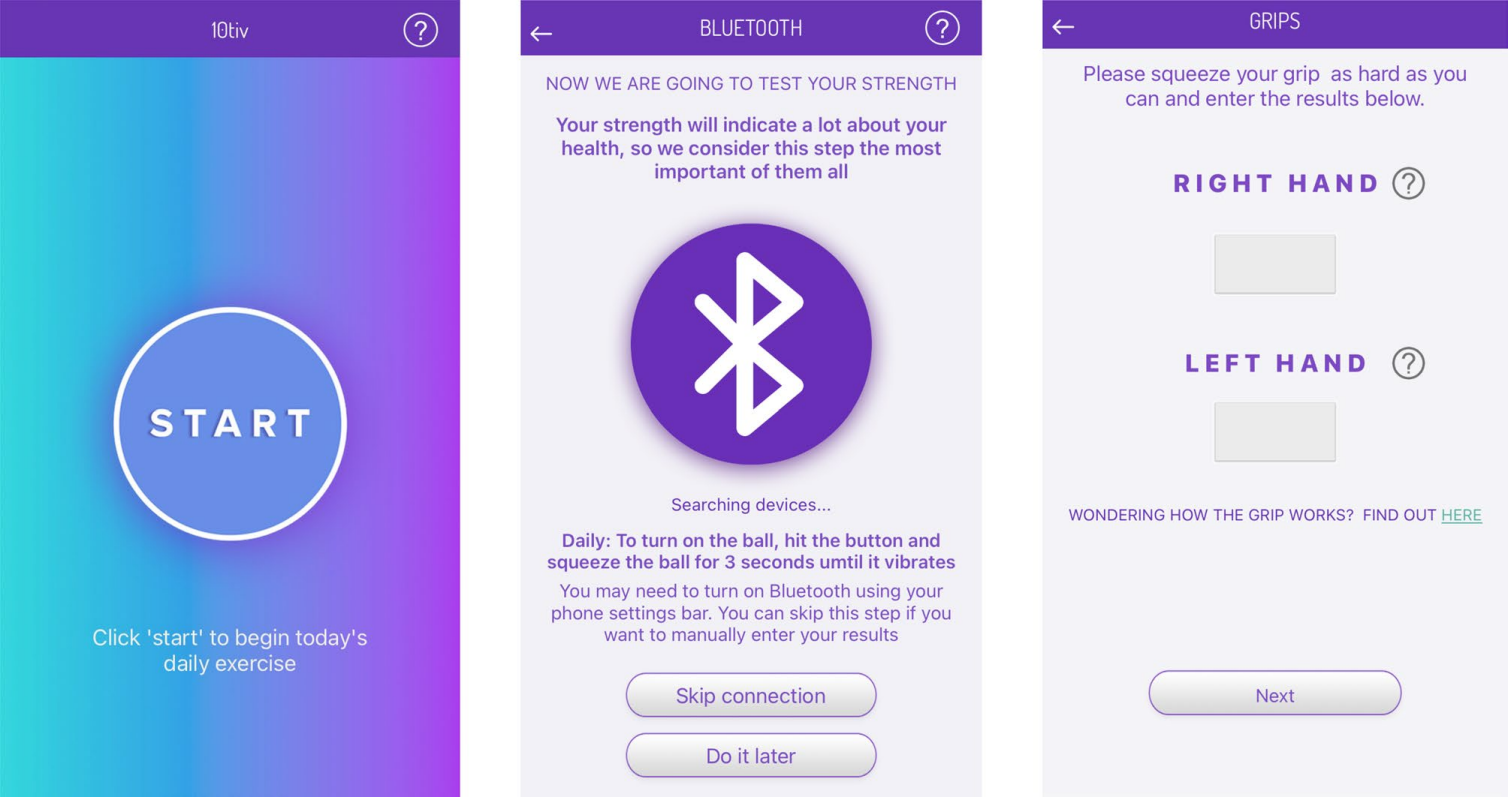
Screenshots of the 10tiv smartphone application displaying the grip strength questionnaire using the squeeze ball dynamometer.

**Figure 3:**
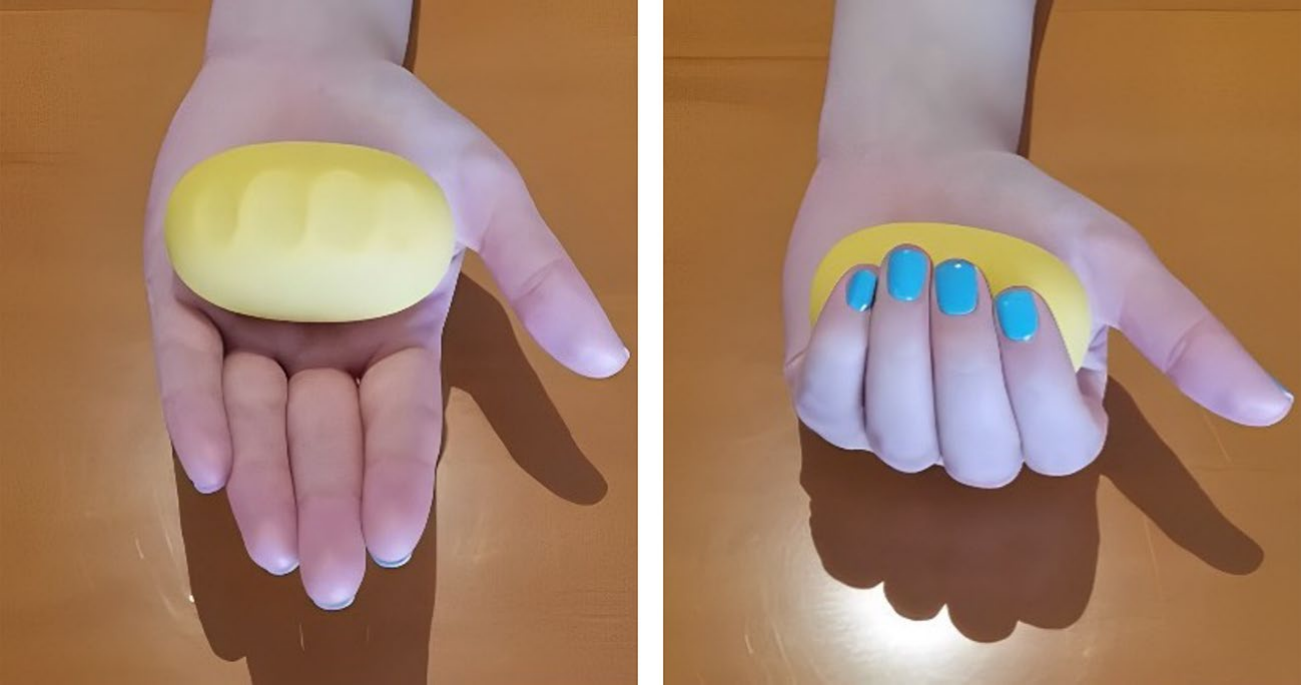
Squeeze ball dynamometer (Smart Stress Ball, 2019 China) used by participants to record daily grip strength to their smartphones via Bluetooth.

Participants were provided with free access to the “10tiv®” smartphone application (Fig. 2, the NTF group, v1.1.6, 2023 Australia) and a squeeze ball dynamometer (Fig. 3, Smart Stress Ball, 2019 China) which connected via Bluetooth to the 10tiv smartphone application. All outcome data submitted by participants were collected through the 10tiv smart phone application and stored in the 10tiv database, located in a secure Australian Amazon Web Services cloud server. No personally identifiable information was stored in the 10tiv database. Access to this database was limited to Australia and the research team using a username and password with two-factor authentication.

At 07:00 pm each day, participants received an automated mobile “push notification” to complete the day’s outcome measures and further automated notifications were sent to participants in the event of missed submissions. The research team actively monitored participant response rates using the 10tiv data dashboard with real-time visibility of data capture and followed up repeated non-completions with a phone call from the trial coordinator. Throughout the trial, participants received weekly phone calls from the trial coordinator during which adverse event reports were collected.

### Experimental procedure

Seven days were allocated to the trial’s run-in phase as participants familiarised themselves with the 10tiv smartphone application and squeeze ball dynamometer. The first four days of the run-in were used as a Pre-baseline practise phase. The final three days of the run-in were used as a Baseline phase, with reported outcome measures across the three days averaged to calculate the Baseline scores against which the intervention was compared (see Fig. 1).

Participants submitted their outcome measures every evening during the trial’s one-week run in, four-week intervention phase and one-week washout. On the first day of the Treatment phase (Day 8), participants commenced application of the transdermal CBD gel three times per day at approximately 08:00 am, 02:00 pm and 08:00 pm. This dose regimen was maintained throughout the Treatment phase (Weeks 2–5).

Participants were instructed to wash their hands prior to each application, thoroughly dry their hands, apply a single pump (0.25 mL) of the transdermal CBD gel to the back of the treatment hand and massage the gel into the skin surrounding the joints until dry. They were also instructed to refrain from washing the treated hand for at least 30-min after application. With regard to the untreated hand, participants were instructed not to apply any CBD gel to this hand and to rinse it thoroughly after each application of CBD gel to the treated hand. It is expected that transdermal absorption of the CBD gel through the palm or fingertips of the untreated hand would be minimal due to the action of rinsing the hand immediately after application.

At the end of the four-week Treatment phase (i.e., on Day 35), participants collected and stored a single urine sample in a freezer until their final, in-person visit with the trial physician. The research team then batched and stored all participant samples at − 20 °C for ~ 3 days until they were transported using dry ice for analysis at the Lambert Initiative for Cannabinoid Therapeutics analytical chemistry laboratories. This aimed to determine whether CBD and its metabolites, 7-hydroxy-cannabidiol (7-OH-CBD) and 7-carboxy-cannabidiol (7-COOH-CBD) could be detected and provided an indication of whether transdermal CBD application led to systemic absorption of CBD.

On the first day of the Washout phase (Day 36, Fig. 1), participants ceased transdermal CBD gel application but continued with the 10tiv-based submission of their outcome measures. At the conclusion of the trial, the trial coordinator completed a follow-up phone call with all participants to discuss their transition out of the trial.

### Outcome measures

#### Primary outcome measure

*Hand Pain.* Subjective measures of hand pain were submitted daily using the Numeric Pain Rating Scale (NPRS), a validated diagnostic tool that measures pain intensity on a 11-point scale (0 (no pain) to 10 (extreme pain))^53,54^. Participants were asked three questions relating to hand pain (1) ‘In your affected joints, how strong is your CURRENT pain?’ (Current pain), (2) ‘In your affected joints, how strong was your AVERAGE pain today?’ (Average pain) and (3) ‘In your affected joints, how strong was your WORST pain today?’ (Maximum pain).

#### Secondary outcome measures

*Grip Strength.* Grip strength is defined as they amount of static force that a hand can generate through the motion of squeezing and provides an indication of functional integrity of the hand^55,56^. A squeeze ball dynamometer (Fig. 2) connected via Bluetooth to the 10tiv smartphone application was used to measure grip strength daily. For patients identifying their thumb as the most painful digit, pinch strength, that is, the use of only the index finger and thumb, was used as an alternative. The 10tiv smartphone application guided participants through a standardised process to capture grip or pinch strength data for both the treated and untreated hands.

*FIHOA.* The Functional Index for Hand OA is a validated questionnaire consisting of ten questions scored on a four-grade scale, allowing for the categorisation of functional impairment from 0 (no functional impairment) to 30 (maximal impairment). This outcome has been used in previous studies of OA^57–59^. In the current study, the FIHOA score was measured weekly throughout the study run-in, intervention phase and washout using the 10tiv smartphone application, allowing for the consideration of the change in hand functionality pre-/post-treatment.

#### Exploratory outcome measures

*Subjective QoL measures.* Subjective QoL Numeric Rating Scales (NRSs), delivered through the 10tiv smartphone application, were measured daily and included fatigue from 0 (‘no unusual fatigue’) to 10 (‘significant unusual fatigue’), stiffness from 0 (‘no stiffness’) to 10 (‘very stiff’), anxiety from 0 (‘not anxious’) to 10 (‘very anxious’) and sleep quality from 0 (‘very disturbed’) to 10 (‘very improved’). These measures were not derived from a validated questionnaire, but rather included to provide simple numerical self-report of subjective troublesome symptoms of hand OA^36^.

*Urinary Cannabinoid Concentrations.* The samples collected from participants on Day 35 were analysed by the Lambert Initiative for Cannabinoid Therapeutics analytical chemistry laboratory to determine urinary CBD, THC and metabolite concentrations (7-COOH-CBD, 7-OH-CBD, 11-OH-THC and 11-COOH-THC) using liquid chromatography-tandem mass spectrometry (LC-MS/MS) applying previous published methods^60,61^. Briefly, urine samples were hydrolysed with β-glucuronidase and analytes subsequently extracted using supported liquid extraction prior to quantification using a Shimadzu LCMS-8040 (Shimadzu Corp., Kyoto, Japan). All samples were analysed in triplicate and quantified against a seven-point standard curve, using deuterated forms of each analyte as internal standards.

### Statistical methods

All data processing and statistical analyses were performed in R v 4.2.3^62^. All analyses included “participant” as the only random factor to account for the repeated measures design. Due to a skewness towards female participants (n = 11/15), gender was not included in any of the analyses to avoid overfitting. All graphs were created using GraphPad Prism v 9.5.1 for Windows (GraphPad Software LLC).

Ordinal variables, namely, Hand Pain NPRS, FIHOA and Subjective QoL NRSs, were analysed by Cumulative Link Mixed Models (CLMM) fitted with the Laplace approximation by the function *clmm* from the package “ordinal”^63^. *P*-values were generated by the type III Wald chi-square test using the function *Anova.clmm* from the package “RVAideMemoire”^64^. Each of these tests initially included Phase (Baseline, Treatment and Washout), time point (day for NPRS and NRS, and week for FIHOA), and their interaction as fixed factors. The models were further refined by removing fixed factors based on the corrected Akaike information criterion (AICc) calculated with the AICc from the package “MuMIn”^65^. We calculated Δm between models and excluded models with Δm > 2 as having substantially less support^66^.

Grip Strength for the treated and untreated hands was analysed separately by two Generalised Linear Mixed Models (GLMM) by the function *glmer* from the package "lme4" version 1.1–30^67^. Residual plots, Shapiro–Wilk test of normality and Pearson's dispersion test were used to identify the best distribution (Gaussian, Gamma, or Inverse Gaussian) and link (Identity, Inverse, Log or Square root) for each model^68^. *P*-values were generated by the type III Wald chi-square test using the function *Anova* from the package “car”^69^.

For all CLMMs and GLMMs, pairwise comparisons by Phase were performed using Dunn-Šidák corrections through the functions *emmeans*, with a time point covariate reduction, and “emtrends” from the package “emmeans”^70^. The threshold for statistical significance was set at α = 0.05.

### Consent for publication

Consent for the images used in Figs. 2 and 3 of this publication have been sought. The persons providing consent have been shown the article contents to be published. All other images are the property of the authors of this article.

## Results

Eighteen participants were consented to the trial but three females were withdrawn by the trial physician prior to the collection of outcome measures due to no longer meeting the eligibility criteria (i.e., ongoing or a history of cannabis-based therapy during the last three months (n = 2) and rheumatoid arthritis diagnosis (n = 1)). A total of 15 participants were included in the final data analysis.

As this is an open label trial with a single treatment arm, an intention to treat analysis (ITT) was not used as there were no data to analyse for the withdrawn participants. ITT analyses are typically used in randomised controlled trails with more than one treatment arm.

On average, the daily participation in submission of outcome measures was 96% with only one participant failing to complete outcome submissions at a level of 90% or above.

### Demographic characteristics

Participant (n = 15) demographic characteristics are summarised in Table 1. The most common characteristics were Australian ethnicity (66.7%), female gender (73.3%), an age between 60 and 69 years old, having a Bachelor’s degree (53.3%), a retired employment status (66.7%) and a managerial (33.3%) or professional (33.3%) occupation.

**Table 1:** Demographic characteristics of participants in this study (n = 15).

Characteristic	N = 15	%
Gender identity		
Male	4	26.7%
Female	11	73.3%
Age		
50–59	1	6.7%
60–69	8	53.3%
70–79	4	26.7%
> 80	1	6.7%
Ethnicity		
Australian	10	66.7%
European	5	33.3%
Grip strength		
Hand^a^	13	86.7%
Thumb^b^	2	13.3%
Education		
Bachelor’s degree	8	53.3%
High school	5	33.3%
Master’s degree	2	13.3%
Employment status		
Retired	10	66.7%
Self-employed	2	13.3%
Part-time employment	1	6.7%
Full-time employment	2	13.3%
Occupation		
Manager	5	33.3%
Clerical and administrative work	2	13.3%
Professionals	5	33.3%
Community and personal services workers	3	20.0%

^a^Participants identifying a finger other than the thumb as the most painful digit; these participants used the whole hand to measure grip strength.

^b^Participants identifying their thumb as the most painful digit; these participants used only the index finger and thumb to measure “pinch” strength (that is, an alternative to grip strength).

### Hand pain

Current hand pain scores were affected by Phase (Baseline, Treatment and Washout) (X^2^_2_ = 6.169, *p* = 0.046) and by the interaction between Phase and Day (X^2^_3_ = 68.320, *p* < 0.0001). Average daily hand pain scores were affected only by the interaction between Phase and Day (X^2^_3_ = 61.870, *p* < 0.0001) and Phase trended towards a significant effect (X^2^_2_ = 5.697, *p* = 0.058). Maximum daily hand pain scores were affected only by the interaction between Phase and Day (X^2^_3_ = 46.000, *p* < 0.0001) but not by Phase alone (X^2^_2_ = 3.742, *p* = 0.154). All hand pain scores were lower during CBD gel application (Treatment phase) than Baseline (Table 2). All hand pain scores were also lower during Washout phases compared to Baseline (Table 2, *p* < 0.0001), and there was no difference between Treatment and Washout phases (Current pain *p* = 0.963, average pain *p* = 0.997 and maximum pain *p* = 0.998).

**Table 2:** Mean (± SEM) numeric pain rating scale scores for current, average and maximum pain during the baseline, treatment and washout phases of the trial.

	Baseline	Treatment	Washout
Current pain mean	5.15 ± 0.23	4.10 ± 0.07	4.20 ± 0.16
Average pain mean	5.45 ± 0.18	4.47 ± 0.07	4.48 ± 0.15
Maximum pain mean	5.57 ± 0.28	4.36 ± 0.10	4.30 ± 0.17

**Figure 4:**
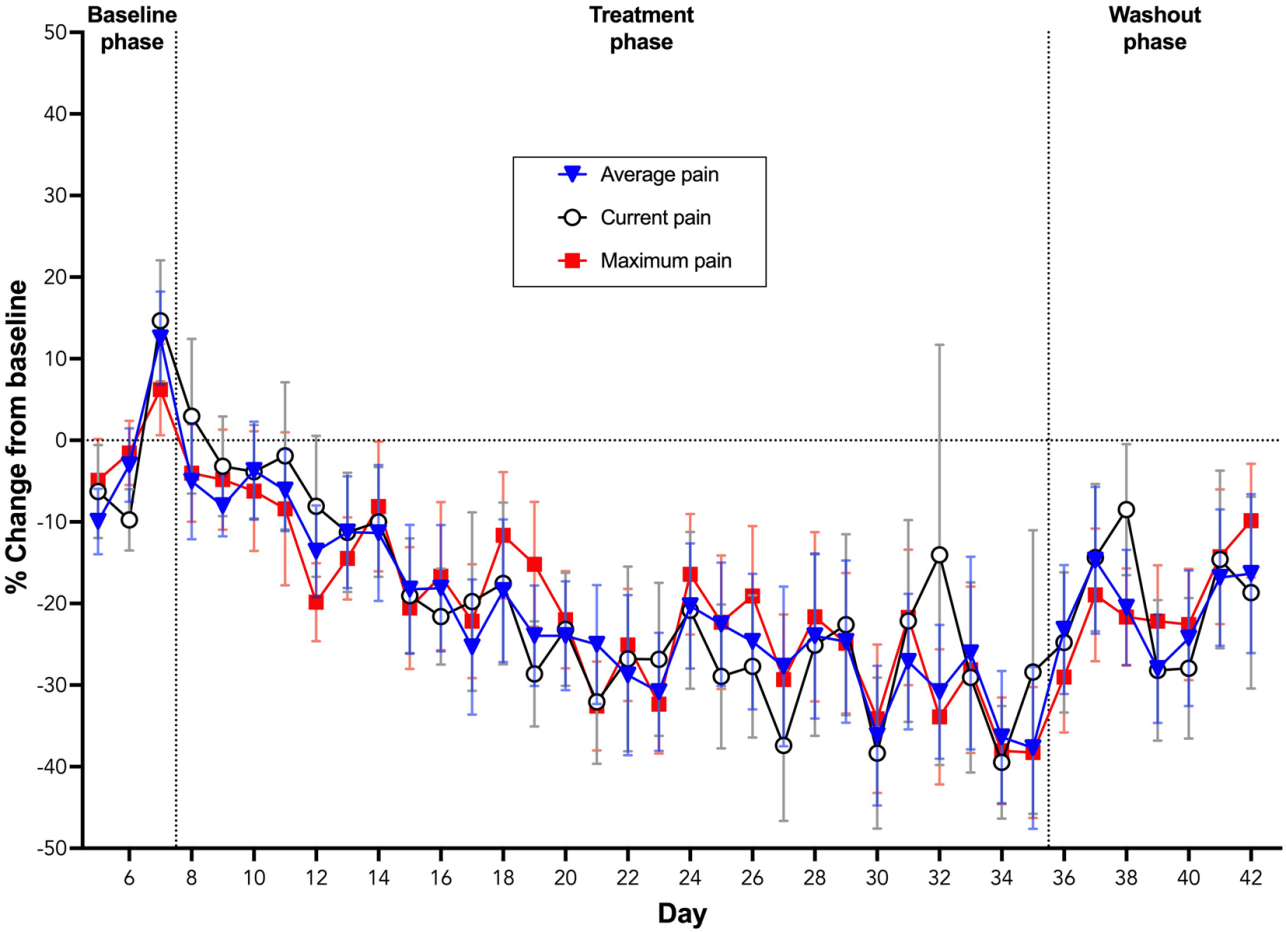
Percentage change from baseline (% ± SEM) for daily hand pain scores (average, current and maximum) during the baseline, treatment and washout phases of the trial.

Current and average daily hand pain scores decreased over time within the Treatment phase (Current pain slope = − 0.098 ± 0.013 and average pain slope = − 0.094 ± 0.013) (mean ± SEM) compared to Baseline (Current pain slope = 0.938 ± 0.396, *p* = 0.027; and average pain slope = 0.873 ± 0.381, *p* = 0.034) (Fig. 4). In contrast, the change over time during the Washout phase (Current pain slope = 0.015 ± 0.105 and average pain slope = 0.014 ± 0.226) was not different from the Baseline phase (*p* = 0.071 and *p* = 0.087, respectively) or Treatment phase (*p* = 0.633 and *p* = 0.672, respectively) (Fig. 4). Maximum daily pain scores demonstrated a decreasing trend over time during the Treatment phase (slope = − 0.080 ± 0.012) and an increasing trend during Baseline (slope = 0.260 ± 0.380) and Washout (slope = 0.143 ± 0.101) (Fig. 4). However, these slopes were not significantly different (*p* > 0.05 for all comparisons).

### Grip strength

Grip strength in the treated hand was affected by Phase (Baseline, Treatment and Washout) (X^2^_2_ = 17.336, *p* < 0.0001), Day (X^2^_1_ = 20.021, *p* < 0.0001) and the interaction between Phase and Day (X^2^_2_ = 39.969, *p* < 0.0001). Grip strength in the untreated hand was not affected by Phase (Baseline, Treatment and Washout) (X^2^_2_ = 0.048, *p* = 0.976), Day (X^2^_1_ = 0.047, *p* = 0.828) or by the interaction between Phase and Day (X^2^_2_ = 0.021, *p* = 0.989).

**Figure 5:**
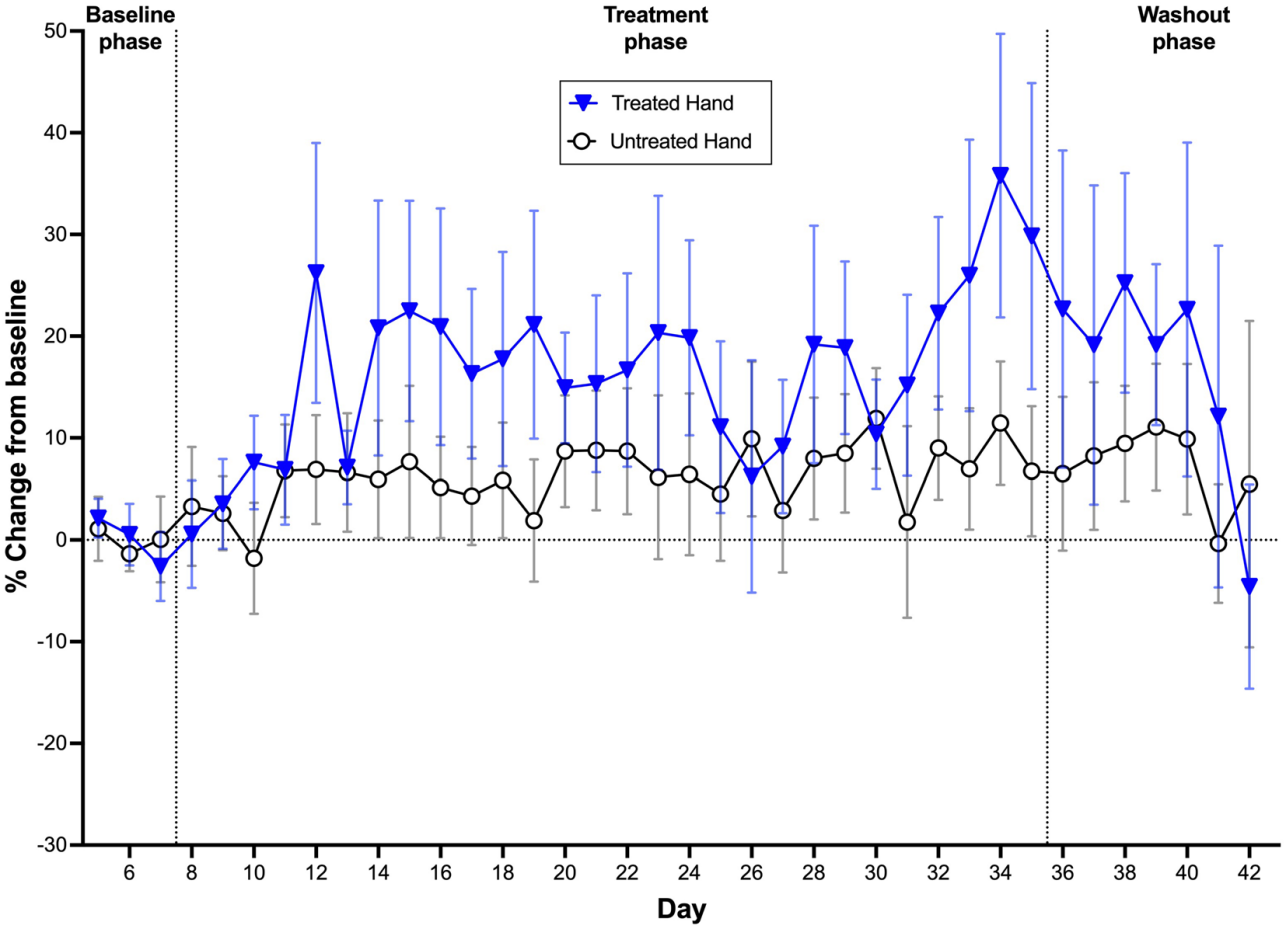
Percentage change from baseline (% ± SEM) for daily grip strength scores of the treated and untreated hands during the baseline, treatment and washout phases of the trial. *Note* the grip strength of the untreated hands is not used as a control and acts only as a reference point.

Pairwise comparisons showed no differences in Grip strength in the treated hand during the Treatment or Washout phases relative to Baseline. However, Grip strength in the treated hand increased over time during the Treatment phase (slope = 0.013 ± 0.004) (mean ± SEM) compared to the Baseline phase (slope = − 0.041 ± 0.009, *p* < 0.0001) (Fig. 5). In contrast, the change over time during the Washout (slope = − 0.022 ± 0.035) was not different from either the Baseline (*p* = 0.942) or Treatment phase (*p* = 0.675) (Fig. 5).

### Functional index for hand osteoarthritis

Participant FIHOA scores were not affected by either Phase (X^2^_2_ = − 2.788, *p* = 1.000) or Week (X^2^_1_ = − 10.098, *p* = 1.000).

### Subjective QoL NRS measures

Fatigue scores were not affected by Day (X^2^_1_ = 1.522, *p* = 0.217) or Phase (X^2^_2_ = 4.869, *p* = 0.088). Sleep scores were affected by Day (X^2^_1_ = 9.985, *p* = 0.002), but not by Phase (X^2^_2_ = 3.831, *p* = 0.147). Stiffness scores were affected by Phase (X^2^_2_ = 15.565, *p* < 0.0001) and Day (X^2^_1_ = 16.919, *p* < 0.0001). Anxiety scores were affected by Phase (X^2^_2_ = 25.093, *p* < 0.0001) and Day (X^2^_1_ = 9.448, *p* = 0.002). Regardless of Phase, sleep scores increased across days (Z = 3.154, *p* = 0.002), while a decrease across days was recorded for stiffness (Z = − 4.086, *p* < 0.0001) and anxiety (Z = − 3.026, *p* = 0.002).

**Figure 6:**
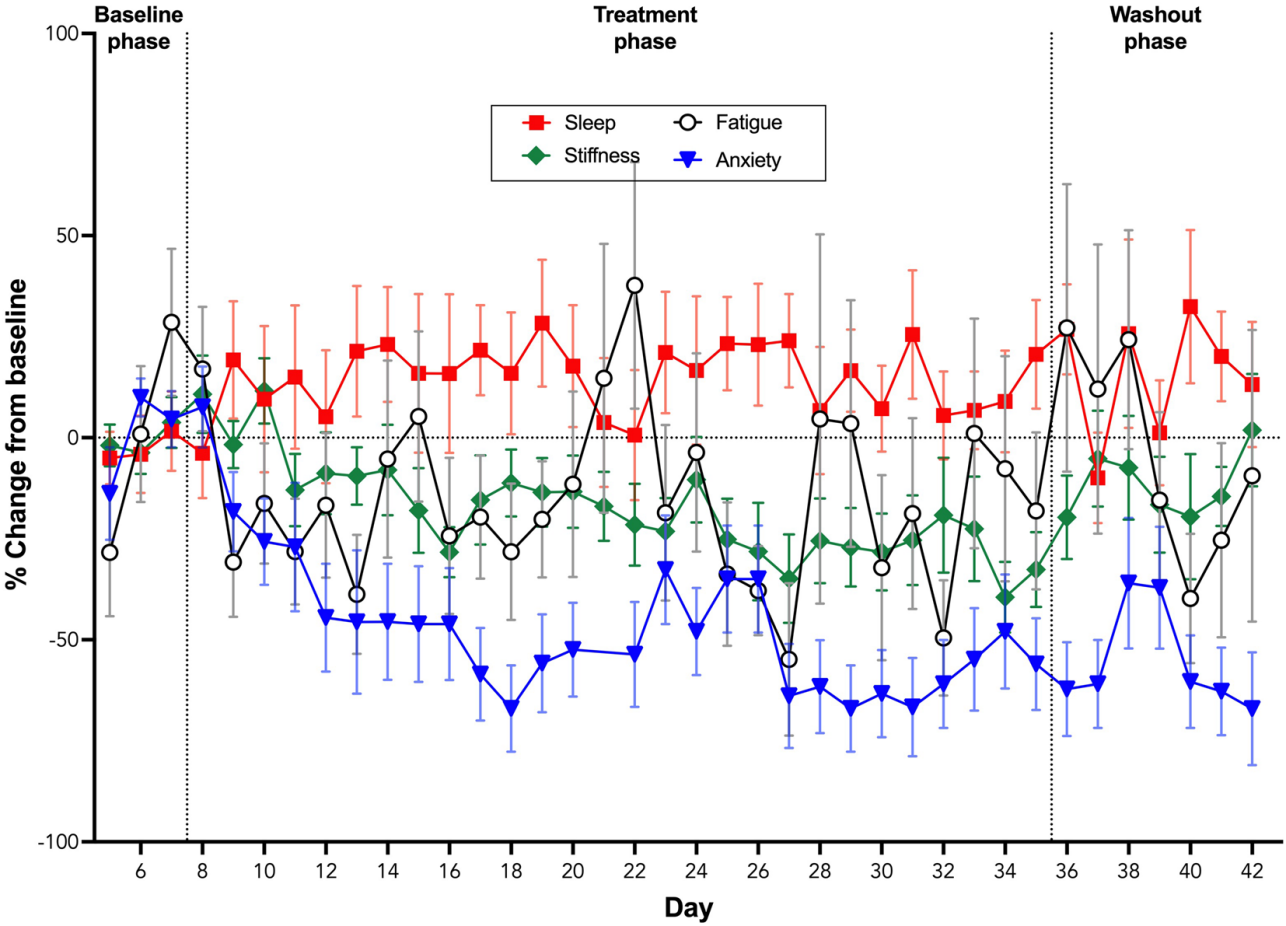
Percentage change from baseline (% ± SEM) for daily self-report of anxiety, fatigue, sleep and stiffness scores during the baseline, treatment and washout phases of the trial.

Overall scores were lower during the Treatment phase for fatigue (*p* = 0.014), stiffness (*p* = 0.004) and anxiety (*p* < 0.0001) (Fig. 6) compared to the Baseline phase. During Washout, scores for fatigue (*p* = 0.009) and anxiety (*p* < 0.0001) remained lower compared to the Baseline phase, while scores for stiffness did not differ compared to either Baseline (*p* = 0.054) or the Treatment phases (*p* = 0.772) (Fig. 6).

### Urinary cannabinoid concentration

**Figure 7:**
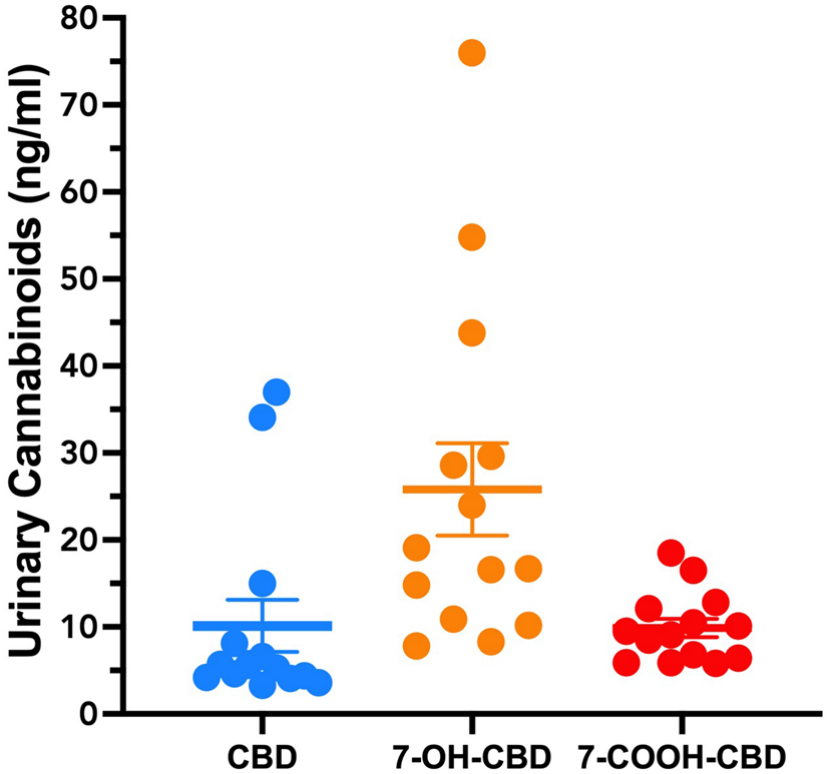
Urinary cannabinoid concentrations of participants (n = 15) at the end of the treatment phase (day 35).

Urine samples taken from participants on the last day of the Treatment phase (day 35, Week 5) with transdermal CBD showed detectable urinary cannabinoids including CBD (10.12 ± 2.89 ng/mL) (mean ± SEM); the primary metabolite 7-OH-CBD (25.79 ± 5.13 ng/mL); and the terminal metabolite 7-COOH-CBD (9.88 ± 1.03 ng/mL) (Fig. 7). There was no detection of THC or THC metabolites in any of the urine samples.

### Adverse effects

During weekly follow-up phone calls with the trial coordinator, a total of 31 mild adverse events (AE) reports were received (‘headache’(n = 6), ‘back pain’(n = 4), ‘neck pain’ (n = 3), ‘shoulder pain’ (n = 3), ‘reflux’ (n = 3), ‘allergy’ (n = 3), ‘pain’ (n = 2), ‘could not sleep’ (n = 2), ‘gastritis’ (n = 1), ‘cramps’ (n = 1), ‘pain in body’ (n = 1), ‘full body pain’ (n = 1) and ‘inflammation’ (n = 1)). One ‘moderate’ AE report was received (‘tooth extraction pain after surgery’). Most of these adverse effects resolved within the day.

## Discussion

The current study explored the effects of a novel, transdermal 4% w/w CBD gel applied three times a day over a four-week treatment phase in patients with symptomatically active hand OA. Innovative features of the trial include the use of a novel smartphone application and Bluetooth squeeze ball dynamometer technology. The primary outcome, namely a significant reduction in hand pain relative to baseline, was met. There was also a significant improvement in hand functionality, measured by grip strength of the treated hand during the Treatment phase compared to the Baseline. However, self-reported hand functionality, measured by the FIHOA questionnaire, was not significantly improved by treatment. Participants in this study also experienced significant improvement in QoL measures relating to anxiety, stiffness and fatigue, but not sleep quality. Systemic absorption of the study drug was confirmed by the presence of CBD and its metabolites in urine samples. Participants demonstrated a high level of proficiency with the technological aspects of the trial.

Self-reported current, average and maximum hand pain were significantly reduced during treatment with transdermal CBD gel. Summary data (Table 2) suggests a somewhat variable reduction in pain across participants. Differences in the structure and integrity of participants skin can affect transdermal absorption^71,72^ and this may partly explain variations in responses to treatment between participants. When normalised by baseline values, however, a ~ 30% reduction in pain scores become evident (Fig. 4). A reduction of approximately 2-points, or 30% in an 11-point NPRS is considered a clinically significant difference^73^. Despite these findings, it is well known that placebo responses contribute significantly to pain reductions seen in many randomised clinical trials of medicinal cannabis and other interventions. Caution is therefore necessary in interpreting these results and placebo-controlled randomised clinical trials will be necessary to give definitive evidence of efficacy^74^.

Urinalysis conducted at the end of the Treatment phase confirmed that CBD achieved some systemic absorption after topical application of the gel. Reductions in anxiety and fatigue measures in participants could conceivably involve central effects of topically applied CBD. Urinary concentrations of CBD and its metabolites were modest however relative to those reported following orally administered CBD (120–480 mg)^75^. The long latency in the return of pain during washout might indicate possible development of a depot of CBD in the stratum corneum of the skin^35^.

The effect of CBD on pain and inflammation is not entirely understood due to the highly complex signalling mechanisms that are engaged by the drug^76^. As well has having indirect modulatory effects on CB1 and CB2 cannabinoid receptors, CBD also affects the serotonin 1A receptor (5‐HT1A), G protein‐coupled receptors 55 (GPR55) and 18 (GPR18) and the transient receptor potential (TRP) vanilloid type 1 (TRPV1) and TRP ankyrin 1 (TRPA1), amongst others^12,21,23^. Preclinical evidence suggests that modulation of TRPV1, TRPA1, CB1 and CB2 receptors provides analgesic and anti-inflammatory effects relevant to the development of OA^25,26,29,30,77^, and that CBD reduces inflammation^78^. It is plausible that CBD provides indirect analgesic effects by moderating synovitis, synovial thickening and effusion in hand OA^79^. Future studies of transdermal CBD would therefore benefit from the additional use of objective measures of hand OA such as X-ray imaging and biomarkers of hand OA to determine possible mechanisms of action or disease modifying effects^80,81^.

Pain is the cardinal driver for treatment-seeking in hand OA and is often used to gauge disease progression and the effectiveness of treatment^82–84^. Damage to an osteoarthritic joint can cause a combination of inflammatory, nociceptive, and neuropathic pain that is generally chronic and difficult to treat^85^. These differing pain phenotypes are suggested in the wide variability of treatment response between patients and may also signal the subjective perception of pain itself^86^. The complication of inadequate pain relief in hand OA is compounded by the limited range of treatment options currently available^36^. Consequently, transdermal CBD may present a novel and safe treatment option for hand OA.

Hand OA leads to a deterioration in grip strength^55^; in one study, women with hand OA had average grip strength measures 60% less than controls^87^. Grip strength is considered a simple, reliable and inexpensive measure of hand function^5,42^. In the current study, there were encouraging improvements in grip strength during the treatment phase. It could be speculated that this improvement related to the daily use of the squeeze ball dynamometer. However, there is only low-quality evidence indicating small beneficial effects of exercise on grip strength in hand OA^87,88^. Furthermore, the grip strength of the untreated hand was not improved during the Treatment phase, despite daily use of the squeeze ball dynamometer.

Despite improved grip strength, there were no improvements in participants’ FIHOA scores. FIHOA requires participants to answer 10 questions around their hand functionality considering a range of activities in the previous week. Notably, some of these queries may not be relevant to all participants. For example, “*For women—*are you able to sew? *For men—*are you able to use a screwdriver?*”* This may distort results and this would be compounded further if the non-dominant hand of participants was used as the treated hand in this study. Notably, most of the participants in this study were over the age of 60 years and female, in line with general population estimates for hand OA^1,36^. The sensitivity of FIHOA in detecting functional improvement in this cohort remains somewhat unclear.

Participants in this study reported significant improvements in subjective measures of anxiety, stiffness and fatigue, but not sleep quality, during the Treatment phase of the study. CBD has demonstrated anxiolytic effects in previous studies; however, these effects are generally seen at oral CBD doses ≥ 300 mg^89–93^. Consequently, it is likely that the reductions in subjective anxiety, and fatigue, are an indirect effect resulting from improved pain outcomes in the current study rather than a direct pharmacological effect of CBD. Similarly, the significant improvements in subjective stiffness may be an indirect effect of the anti-inflammatory properties of CBD^21,23–26,28–30,94^ which in turn could be responsible for the objective increase in grip strength. The improvements in pain outcomes in the current study did not appear to improve sleep.

Significant limitations in the current study should be acknowledged: this was a non-randomised, interventional, feasibility trial involving a small number of participants. A significant placebo effect is seen in many randomised controlled trials of interventions for chronic pain and the lack of placebo is therefore a significant limitation. No blood samples were taken during the study limiting our understanding of the pharmacokinetics of the study drug. Positive aspects of the current study include use of novel smartphone technology that was secure and instantaneous, and led to excellent rates of compliance and retention in study participants. This internet-based study provided a cost-effective and convenient methodology for participants with varying levels of functional impairment due to their hand OA, benefits that have been demonstrated in other internet and/or smartphone application-based studies of hand OA^84,95^.

## Conclusion

The current study suggests that transdermal CBD gel may have a beneficial effect on pain and grip strength in participants with symptomatic hand OA but requires further exploration in a randomised controlled trial. There has been an urgent call for proof-of-concept trials, including those with negative results, to help illuminate the pathogenic mechanisms of hand OA^5^. This pilot study contributes towards closing this evidence gap and demonstrates that transdermal CBD may provide some promise for a safe treatment option for symptomatically active hand OA. Further research should incorporate a double blind, randomised study design with greater participant numbers and more comprehensive pharmacokinetic and biomarker analysis.

## Data Availability

The trial data are available from the corresponding author on reasonable request.
